# Inference on spatiotemporal dynamics for coupled biological populations

**DOI:** 10.1098/rsif.2024.0217

**Published:** 2024-07-10

**Authors:** Jifan Li, Edward L. Ionides, Aaron A. King, Mercedes Pascual, Ning Ning

**Affiliations:** ^1^ Department of Statistics, Texas A&M University, College Station, TX 77843, USA; ^2^ Department of Statistics, University of Michigan, Ann Arbor, MI 48109, USA; ^3^ Department of Ecology & Evolutionary Biology, University of Michigan, Ann Arbor, MI 48109, USA; ^4^ Center for the Study of Complex Systems, University of Michigan, Ann Arbor, MI 48109, USA; ^5^ Santa Fe Institute, Santa Fe, NM 87501, USA; ^6^ Departments of Biology and Environmental Studies, New York University, NY 10012, USA

**Keywords:** Markov process, block particle filter, metapopulation model, ecology, epidemiology

## Abstract

Mathematical models in ecology and epidemiology must be consistent with observed data in order to generate reliable knowledge and evidence-based policy. Metapopulation systems, which consist of a network of connected sub-populations, pose technical challenges in statistical inference owing to nonlinear, stochastic interactions. Numerical difficulties encountered in conducting inference can obstruct the core scientific questions concerning the link between the mathematical models and the data. Recently, an algorithm has been proposed that enables computationally tractable likelihood-based inference for high-dimensional partially observed stochastic dynamic models of metapopulation systems. We use this algorithm to build a statistically principled data analysis workflow for metapopulation systems. Via a case study of COVID-19, we show how this workflow addresses the limitations of previous approaches. The COVID-19 pandemic provides a situation where mathematical models and their policy implications are widely visible, and we revisit an influential metapopulation model used to inform basic epidemiological understanding early in the pandemic. Our methods support self-critical data analysis, enabling us to identify and address model weaknesses, leading to a new model with substantially improved statistical fit and parameter identifiability. Our results suggest that the lockdown initiated on 23 January 2020 in China was more effective than previously thought.

## 1. Introduction

Biological populations may be structured into a collection of densely populated communities separated by sparsely populated regions. The network of communities, which may be cities in a human context, comprise a metapopulation. Motivation for metapopulation modelling arises when some essential feature of the population dynamics cannot be understood from looking at a single location. The dynamics of persistence through local extinctions and reintroductions have been extensively studied in ecology [1,2]. In epidemiology, metapopulation dynamics can be a barrier to the regional elimination and eventual eradication of a pathogen and may determine the successful invasion of a new pathogen or a new strain of an existing pathogen [3]. In other situations, spatiotemporal dynamics may be an unavoidable component of the system under study without being the focus of the investigation [4,5].

Mathematical models for biological systems are used to inform public policy, despite delicate issues in the implementation and interpretation of such models [6]. Indeed, it can be practically impossible to make sense of the nonlinear stochastic interactions driving biological dynamics without representing them in a model [7,8]. Difficulties arising in developing these models can be categorized as operational or interpretive. Operationally, we seek to fit complex models using statistically valid, reproducible and transparent methods. Interpretive difficulties arise when drawing causal conclusions from fitting models to observational data, giving rise to opportunities for incorrect conclusions owing to missing variables or other forms of model misspecification. When addressing metapopulation dynamics, the network structure creates the operational challenge of working with systems that have high dimensions in addition to nonlinearity, stochasticity and incomplete observability. The modelling assumptions required to describe movement between sub-populations present additional possibilities for model misspecification.

In this article, we develop a data analysis workflow which remedies some limitations of existing methods. Previous approaches had difficulties carrying out likelihood-based inference because existing algorithms required problematic approximations in order to be computationally feasible. Our approach builds on a recent algorithmic development for evaluating and maximizing the likelihood function for metapopulation models. This development enables the use of likelihood-based methods for parameter estimation, confidence interval construction, model selection and the diagnosis of model misspecification. These likelihood-based inference tools are fundamental statistical techniques, known to have favourable theoretical and practical properties, and so they provide a principled framework for data analysis. The inferential toolkit we present has previously been practical only for low-dimensional systems. A concrete demonstration of our workflow, and its relationship to previous approaches, are carried out via an extended example. We then generalize the lessons learned from this example to provide advice for a practical and methodologically principled inference for metapopulation models.

The recent growth in the study of metapopulation dynamics has been driven partly by the COVID-19 pandemic [9–17] and in part by methodological advances facilitating the fitting of metapopulation models to spatiotemporal data. Until the start of this millennium, developing dynamic models with both statistical and scientific justification was a long-standing open problem for even a single population [18]. Over the past two decades, new algorithms [19–22] and software [23–25], together with ever-increasing computational resources, have enabled routine inference for low-dimensional nonlinear partially observed stochastic dynamic systems. However, fundamental algorithmic scalability issues known as the ‘curse of dimensionality’ lead to difficulties with the high-dimensional systems arising in metapopulation inference. These issues are clearest for Monte Carlo techniques based on importance sampling [26] but are also evident in the need for variational approximations for large Markov chain Monte Carlo (MCMC) calculations [27]. Thus, the data analysis for metapopulation models has lagged behind the analysis of low-dimensional time-series data for biological dynamics. Recent developments enable this gap to be closed, as we demonstrate via a reanalysis of COVID-19, viewed from the context of the ability to draw evidence-based scientific conclusions about the dynamics of the emerging pandemic in January and February 2020.

Biological systems are characterized by nonlinear stochastic dynamics together with incomplete and noisy measurements [18]. We, therefore, focus on the class of partially observed Markov process (POMP) models [28], acknowledging that deterministic models can be conceptually useful but are problematic as statistical explanations of noisy systems [5,29]. The Markov property asserts that the dynamic process has no memory conditional on its current state, which is algorithmically convenient while being scientifically non-restrictive since we can choose what to include in the state. Metapopulation models consider a multivariate system state at each location, and so we require methods tailored for high-dimensional POMP models. Simplifications arise if models and data are limited to binary presence or absence, or a small discrete set of values at each location [2], but we are concerned with situations where time series of abundance data are available, such as case reports for infectious diseases. We focus on two inferential approaches for high-dimensional POMP models, the block particle filter (BPF) and the ensemble Kalman filter (EnKF). Other alternatives are reviewed in electronic supplementary material, section S3.

EnKF was developed in the context of massive geophysical models. It combines an ensemble representation of the latent state with a computationally efficient update rule inspired by the scalable linear Kalman filter, providing an approach with excellent scalability [30,31]. For biological systems, the EnKF was first demonstrated as a computationally convenient tool for compartment models at a single location [32,33]. Subsequently, it has been applied for epidemiological metapopulation inference [9,34]. However, the linearization in the EnKF update rule can be problematic for highly nonlinear systems [31,35]. Furthermore, a linear update rule is not appropriate for small, discrete populations unless the EnKF is embedded within an MCMC algorithm [36]. In contrast, particle filter methods [37] avoid linearization and are directly applicable to discrete and continuous latent states.

For low-dimensional systems, particle filter methods are broadly applicable; they permit consideration of arbitrary nonlinear dynamics and require the model to be specified only via a simulator [23,28]. Particle filters enable statistically efficient use of data, since they provide an evaluation of the likelihood function required for Bayesian or likelihood-based inference, with approximation resulting only from the finite Monte Carlo effort. For high-dimensional systems, scalability considerations demand further approximations since particle filters suffer acutely from the ‘curse of dimensionality’ [26]. The BPF algorithm modifies the particle filter to achieve scalability by carrying out local resampling on spatial neighbourhoods known as blocks. This avoids the linear update rule used by the EnKF [38]. It is an empirical question whether the different approximations inherent in the EnKF and BPF are successful on metapopulation models, with prior evidence favouring the BPF [35]. In the following example, we demonstrate that the BPF can be effective for a practical metapopulation data analysis. We show that the resulting likelihood-based inference framework provides opportunities for model criticism, leading to rigorous assessment of model fit and improved advice on public policy decisions.

## 2. Metapopulation analysis of COVID-19 spread in China

We reconsider the influential analysis of COVID-19 by Li *et al*. [9], from early in the pandemic. This analysis provided estimates of transmission parameters and the effect of the lockdown in China using the limited data available at the time. Other teams have fitted models to address similar questions [16,39,40] but the study by Li *et al.* [9] is distinctive for fitting a stochastic mechanistic metapopulation model to extensive spatiotemporal data. The results were published in May 2020 [6], based on reported cases from 10 January to 8 February of that year. The state-of-the-art spatiotemporal analysis was possible on an urgent timescale because the team of researchers had developed their methodology in a sequence of previous situations [32,33,41,42]. The paper is written with attention to reproducibility, and the main results are strengthened by various supporting analyses in an extensive supplement. We show that the approach developed in this article, using algorithms that were not available to Li *et al.* [9], can identify and fix various limitations in their model and inferences. Our goal is not to criticize any specific paper, but rather to build on the timely analysis of Li *et al.* [9] to demonstrate how recently developed techniques provide possibilities to carry out improved data analysis in the future.

**Figure 1.**
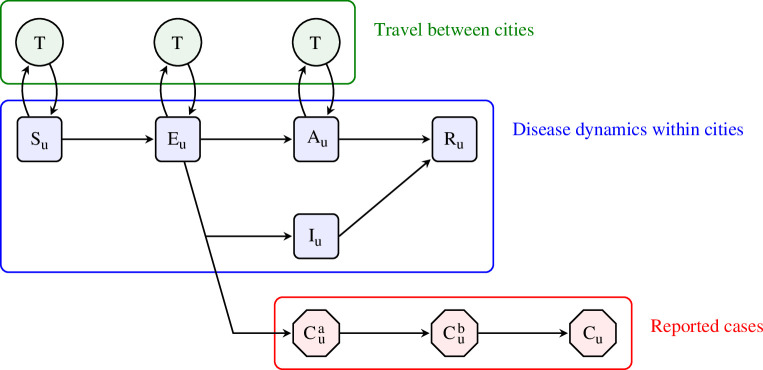
A flow diagram for the SEAIR metapopulation model. Each individual in city u is a member of exactly one of the square blue compartments. Individuals entering the reportable infectious compartment, ${\text{I}}_{u}$ for city u, are simultaneously included in the delayed reporting process compartment, ${\text{C}}_{\text{u}}^{\text{a}}$. Upon arrival at the final reporting compartment, ${\text{C}}_{u}$, the individual is included in the case report for city u. Individuals in ${\text{A}}_{u}$ are not reportable and transmit at a reduced rate. The movement of individuals between cities occurs by transport to and from a transport compartment, T. The number of individuals moving between each pair of cities is based on the 2018 data from Tencent. The movement is modelled only for susceptible, exposed and undetected infections.

For our metapopulation system, the sub-populations are 373 provincial cities in China (meaning cities with administrative responsibility for an entire region) and the data are daily reported COVID-19 cases. COVID-19 dynamics are represented by a susceptible–exposed–asymptomatic–infectious–recovered (SEAIR) epidemic model. Questions of urgent interest early in the pandemic included the relative transmissibility of reported to unreported cases, the fraction of unreported cases and the effect on transmission of movement restrictions imposed on and around 23 January [9]. The model structure is illustrated by the flow diagram in figure 1. Section 4 provides an additional description, with equation (4.1) giving the modelled rate at which susceptible individuals become infected. The complete model specification and estimated parameter values are in electronic supplementary material, section S1.

We consider different model implementations within this structure. Our starting point is model *M*_1_ which is based on the model of [9] and is described in electronic supplementary material, section S1.3. We consider the full dataset, from 10 January to 8 February, with transmission parameters re-estimated following the lockdown on 23 January; these correspond to the periods 1 (10 January to 22 January) and 3 (24 January to 8 February) of [9]. Some minor differences between *M*_1_ and [9] were introduced to enable us to place their model within the general framework of spatiotemporal partially observed continuous-time Markov process models described by Ionides *et al.* [35]. Despite these modifications, simulations from *M*_1_, using the parameters of [9], closely match simulations from the code provided by Li *et al.* [9] (electronic supplementary material, section S1.3). In *M*_1_, the modelled movement rate between cities is proportional to mobility data based on cell phone records from Tencent. However, an inspection of the mobility data reveals that some small cities had no recorded incoming travellers, and therefore, no possibility of introducing SARS-CoV2 within *M*_1_ (or the model of [9]) (electronic supplementary material, section S1.2). This minor limitation formally results in a likelihood of 0 for *M*_1_ (i.e. it is impossible for the simulation model to reproduce the observed spatiotemporal dataset), and hence a log-likelihood of −∞.

We addressed the problematic mobility data in *M*_1_ by adding some additional transportation based on a gravity movement model, as described in electronic supplementary material, section S1.2, giving rise to model *M*_2_. We implemented an additional adjustment between models *M*_1_ and *M*_2_ to align the measurement model with the EnKF inference method presented by Li *et al.* [9]. The EnKF implementation involved specifying a quantity called the observation error variance, defined as a function of the observed cases, to quantify the uncertainty in the measurements. Within the POMP specification, the measurement variance can depend on the latent state but not directly on the observed data. The use of a data-dependent observation error variance is, therefore, inconsistent with the theoretical motivation of the EnKF algorithm as a filter for a POMP model. To interpret the EnKF observation variance chosen by Li *et al.* [9] within the POMP framework, we specified the measurement model for *M*_2_ to have equivalent scaling to the choice of Li *et al.* [9], but with the dependence on the reported cases replaced by dependence on the modelled, but unobserved, exact case count.

Based on a comparison of various nonlinear spatiotemporal filters (electronic supplementary material, figure S7) we evaluated the log-likelihood for *M*_2_ using a BPF (table 1). To account for model overfitting, the number of estimated parameters can be subtracted from the log-likelihood to obtain a comparison equivalent to Akaike’s information criterion (AIC) [43]. When the difference in log-likelihood is large compared to the difference in degrees of freedom, the ordering of statistical goodness-of-fit is clear without presenting formal statistical hypothesis tests.

**Table 1.** Model comparisons by log-likelihood, evaluated by a block particle filter. The degrees of freedom (d.f.) is the number of estimated parameters.

model	log-likelihood	d.f.	description
*M*_1_	−∞	11	SEAIR model using the parameter values and mobility data of [9]
*M_2_*	−14 985.0	11	adjusted mobility and measurement in *M*_1_
*M*_3_	−11 257.9	374	independent identically distributed negative binomial
*M*_4_	−10 825.3	375	autoregressive negative binomial
*M*_5_	−9088.2	15	adding overdispersed dynamics to *M*_2_ and refitting
*M*_6_	−9116.5	13	latent and infectious durations unchanged by lockdown in *M*_5_.

To find out whether this log-likelihood value suggests that the model is satisfactory, we compare it with two simple statistical models: *M*_3_ simply models the daily case report for each city as an independent identically distributed (IID) negative binomial random variable; *M*_4_ adds an autoregressive component to *M*_3_ (see electronic supplementary material, section S2). We see from table 1 that both *M*_3_ and *M*_4_ outperform *M*_2_ by many units of log-likelihood. Likelihood can properly be compared between different models for the same data, with statistical uncertainty in log-likelihood differences arising on the unit scale [44]. When the fit of a mechanistic model is inferior to a simple statistical model, we learn that the mechanistic model has room for improvement as a description of the data, but we do not immediately learn what the deficiency is. The development of methods for formal statistical fitting of mechanistic models has led to an increased understanding of the importance of appropriate modelling of over-dispersed variation in stochastic dynamics [45–47]. We, therefore, hypothesized that the fit of *M*_2_ could be improved by permitting additional dynamic noise.

A standard way to convert a deterministic model, constructed as a system of ordinary differential equations, into a stochastic model is to reinterpret the rates of the deterministic system as rates of a Markov chain [48]. This places limits on the mean–variance relationship of the resulting stochastic model [49]. Models allowing greater variability than permitted by this construction are said to be over-dispersed. We added multiplicative white noise to the transmission rate, following the approach of [28,45], giving rise to model *M*_5_. We fitted the model using an iterated BPF to maximize the likelihood [50,51]. The block filter approximation has also proven helpful for spatiotemporal inference when using alternatives to particle filtering and alternatives to maximization by iterated filtering [47]. In the current context, the BPF was found to be more effective for likelihood evaluation than a test suite of alternative filters including the EnKf (electronic supplementary material, figure S7). The iterated BPF (IBPF) maximizes the BPF likelihood using an iterated filtering algorithm [22] adapted to the structure of a BPF.

**Figure 2.**
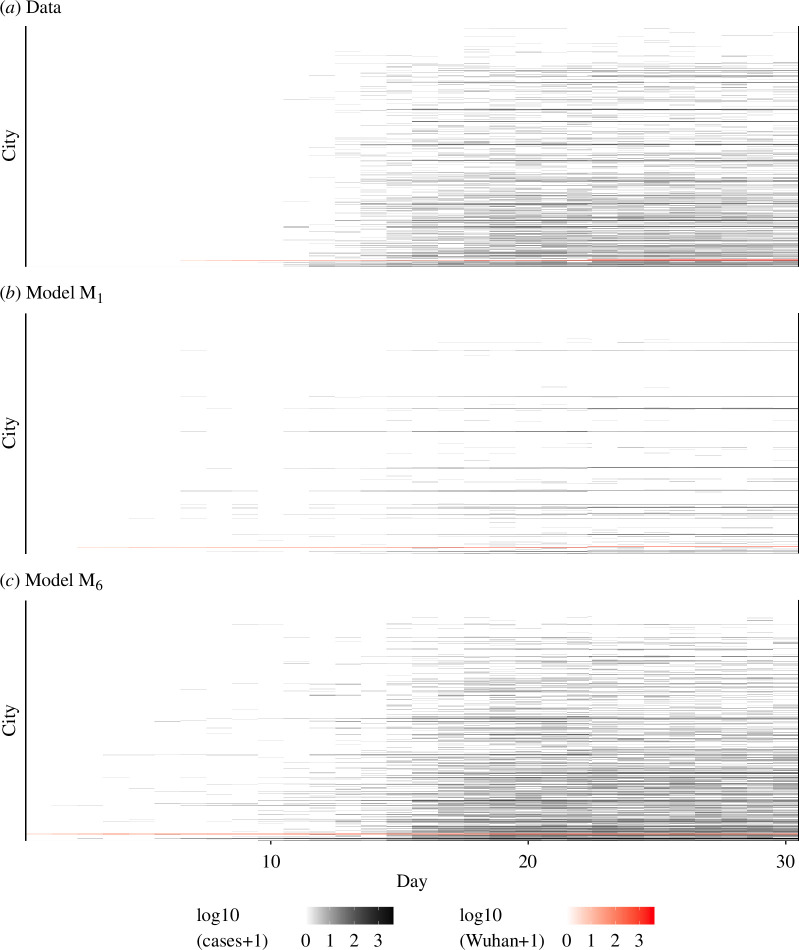
Daily case report time series for 373 cities: (*a*) the real data; (*b*) a simulation from model M_1_; (*c*) a simulation from model M_6_. Within each panel, cities are ordered by population, the largest being on the bottom row.

Table 1 shows that model *M*_5_ outperforms simple statistical benchmarks, obtaining a competitive likelihood with relatively few parameters. From a statistical perspective, *M*_5_ is, therefore, an adequate statistical description of the data. However, some parameters of *M*_5_ were weakly identified by the data, especially in the pre-lockdown time interval within which there were relatively few reported cases (electronic supplementary material, section S6). When the evidence about the model parameters in the data is weak, the ambiguity may be resolved by other, unmodelled and poorly understood, aspects of the data. This risks leading to undesirable situations where important conclusions about questions of interest could be driven by the weaknesses of the model rather than its strengths. In electronic supplementary material, section S6, we show how the flexibility of *M*_5_ can be used to obtain a high likelihood via an implausibly long estimated duration of infection during the pre-lockdown period, with the estimate suddenly reducing after lockdown. We resolved this issue by constraining the latent and infectious periods to be the same before and after lockdown, leading to model *M*_6_. A time-varying effective infectious period could be appropriate owing to changes in quarantine practices, so we let the data assess whether a constant infectious period is plausible in the context of this model. The additional constraints of *M*_6_ lead to a small loss of likelihood compared to *M*_5_, but the fit remains competitive compared to the benchmark models, and the stronger identifiability facilitates the interpretation of estimated parameters. Calculating the maximized log-likelihood for each model in table 1 requires extensive computation to produce a single number which contains essentially all the information about the statistical fit of the model. However, deeper investigation is required to understand what characteristics of the models and data cause the differences in these numbers, and the practical consequences of the numerical results. As a starting point, figure 2 plots the data next to simulations from models *M*_1_ and *M*_6_. Visually, the comparison confirms *M*_6_ as a reasonable representation of the data. Both *M*_1_ and *M*_6_ overestimate cases before day 14, but the context of rapidly increasing awareness and growing diagnostic capabilities is hard to quantify. A visualization of the entire spatiotemporal dataset cannot provide high resolution on specific features of interest, and so this plot is complemented by various other graphical representations in the supplement.

Parameter values for models *M*_1_, *M*_5_ and *M*_6_ are reported in electronic supplementary material, table S1. Here, we discuss the estimated basic reproductive number (i.e. the expected number of secondary infections from one index case in a fully susceptible population), denoted by $R_{0}^{\text{be}}$ and $R_{0}^{\text{af}}$ before and after the 23 January lockdown. $R_{0}$ is calculated by the formula in equation (4.2). Our estimates for model *M*_6_ are $R_{0}^{\text{be}}=3.51$ with confidence interval (CI) (3.31,3.72) and $R_{0}^{\text{af}}=0.70$ with CI (0.65,0.77), where the estimates and their associated 95% CIs are obtained by profile likelihood (electronic supplementary material, section S7). This implies that the Chinese government’s non-pharmaceutical interventions instituted on and around 23 January reduced $R_{0}$ by a factor of 5.0. In contrast, the estimates of [9] are ${\overset{˜}{R_{\text{0}}}}^{\text{be}}=2.38$ with CI (2.03,2.77) and ${\overset{˜}{R_{\text{0}}}}^{\text{af}}=0.98$ with CI (0.83,1.16), implying reduction by a factor of 2.4. For comparison, interventions implemented across a panel of 41 countries (34 European) were estimated to reduce $R_{0}$ by a factor of 4.3 with CI (2.9,6.7) [52]. Our estimate for $R_{0}$ before lockdown is towards the high end of previous estimates based on data up to February 2020, reviewed by Park *et al.* [53]. An alternative metapopulation analysis of the pre-lockdown China data, with a deterministic transmission model, obtained an $R_{0}$ estimate of 3.11 with CI (2.39,4.13) [54]. Our $R_{0}$ estimate is consistent with pre-lockdown estimates from other locations, such as New York City, for models that include asymptomatics [55].

The likelihood-based confidence intervals for *M*_6_ are narrower than the intervals from [9]. However, *M*_6_ fits two fewer parameters than *M*_5_, and the latter is more directly comparable to the model of [9]. For *M*_5_, the likelihood-based analysis leads to wide CIs for some parameters, revealing the weakly identified parameters.

Our model inherits the property of [9] that infections arising during the pre-lockdown period will generally be reported during the lockdown, owing to the reporting delay modelled as a distributed delay with a mean of 9 days pre-lockdown and 6 days post-lockdown. Thus, the model is permitted to explain the data by inferring rapid, unreported spread prior to 23 January. Despite this shared constraint on the form of the model, conclusions of our analysis differ from [9]. Beyond the estimates of $R_{0}$, a notable difference is that we find the estimated transmissibility of observed cases is close to that of unobserved cases, especially before lockdown (electronic supplementary material, table S1).

If a mechanistic model has a likelihood competitive with statistical benchmarks, it is anticipated to have simulations that are qualitatively comparable to the data. Since the model specification is inevitably imperfect and is accounted for in the model fitting by noise processes, we expect simulations from the fitted model to have somewhat more stochastic variation than the data. In contrast, models that are structurally unable to provide sufficient variability to explain the data must give rise to simulations with too little stochasticity (as shown in figure 2). Models that have simulations with implausibly little variability give rise to claims of excessive confidence about the uncertainty surrounding estimated parameters. This phenomenon may be clearest when CIs are calculated using the parametric bootstrap approach, involving re-estimation of parameters using artificial datasets simulated from a fitted model. However, it also applies to classical CI and Bayesian credible interval constructions. Thus, CIs from mechanistic models that outperform statistical benchmarks are anticipated to be conservative, whereas CIs from models with insufficient variability to explain the data are generally anti-conservative. Requiring model likelihoods to be comparable to statistical benchmarks, therefore, improves the credibility of uncertainty intervals as well as improving the accuracy of point estimates.

## 3. Discussion

**Figure 3.**
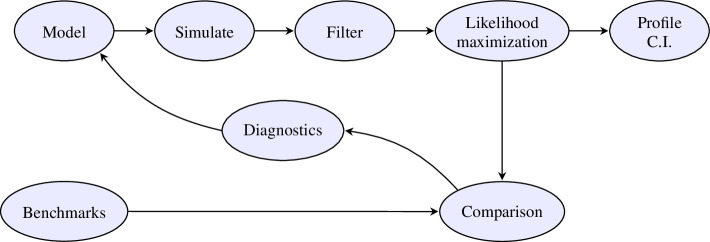
A workflow for likelihood-based data analysis of partially observed spatiotemporal systems. Electronic supplementary material, section S10 provides an additional description of the steps in this workflow.

Advances in statistical methodology will drive an increase in the number of spatiotemporal models fitted to epidemiological data. Our research demonstrates that techniques proven effective in low-dimensional systems, such as population dynamics at one or two locations, can be extended to address larger metapopulation systems. This extension allows us to leverage well-established best practices from time-series analysis, leading to a statistically principled approach. This approach enables us to identify and rectify model limitations that might otherwise remain undetected. A failure to address these weaknesses can lead to issues of irreproducibility and the provision of suboptimal policy recommendations when developing models for complex dynamic systems [6,56]. Principles of good data analysis for population dynamics are presumably similar to the general principles of data science [57] but require some adaptation to the specific situation. Here, we build on earlier analyses [6,56,57], by demonstrating the feasibility and desirability of metapopulation analysis meeting the specific set of criteria outlined below. Our implementation of these criteria is summarized in the data analysis workflow shown in figure 3.

— *Likelihood-based statistical inference*: a model, in conjunction with data, defines a likelihood function that quantifies the goodness of fit of the model and the data for each parameter value. For mechanistic models, it is usually impossible to write down the likelihood explicitly, but it still exists implicitly. Likelihood-based methods can extract all available information in the data about model parameters [44]. Log-likelihood is also a proper scoring rule for comparing probabilistic forecasts [58] and, therefore, provides a sensitive objective tool for model selection and identification of model misspecification. Whereas cross-validation and out-of-sample fit are standard benchmarks in machine learning settings [57], likelihood is better suited to situations with relatively small, spatiotemporally structured datasets. Likelihood-based inferences via particle filters have been inaccessible for metapopulation models owing to the ‘curse of dimensionality’ [26]. However, BPF methods can be effective on metapopulation models, as demonstrated in this paper and previously [5,35,51]. All high-dimensional nonlinear filters entail numerical approximation, and these can be assessed by comparing predictive skill (i.e. the estimated log-likelihood) between different filters. The EnKF provides a suitable point of comparison, since it has excellent scalability properties, modest capability to handle nonlinearities, and has been demonstrated on various epidemiological systems [13,16,32–34,41,42].
— *Statistical benchmarks*: the challenge of fitting intricate nonlinear models to extensive datasets makes it difficult for researchers to evaluate the limitations of their models and methods. Readers also can struggle to determine whether the proposed model has been adequately tested. It is, therefore, advisable to incorporate benchmarks for evaluating model performance in comparison with relatively simple statistical models [45]. This approach helps determine whether complex models provide a satisfactory level of explanatory power. In the first instance, these benchmarks can be applied to the entire dataset; subsequent analysis can focus on dissecting the contributions from various subsets of the data to gain a comprehensive understanding of which parts of the data drive the overall assessment. The likelihood provides a suitable quantity for comparison between different models for the same data [44]. If we find a simple statistical model with a log-likelihood many units higher than a mechanistic model, we have discovered that the mechanistic model is unable to explain some substantial aspects of the data. At the very least, such a discrepancy should be identified and discussed.
— *Residual analysis*: introductory statistics classes, when covering linear regression, emphasize that a careful and complete data analysis involves examining deviations from the fitted model [59]. This is typically achieved by plotting residuals, a suitably rescaled measure of disparities between each observation and its corresponding fitted value. A relevant measure of residual in the current context is the log-likelihood anomaly, defined as the discrepancy between the mechanistic fit and a benchmark for components of the likelihood at each observation. Electronic supplementary material, section S8 describes how these diagnostic tools were used for developing and evaluating model *M*_6_.
— *Uncertainty*: reliable conclusions should be robust to plausible variations in data, models and algorithms [57]. Standard statistical methods provide measures of uncertainty and the validity of these measures depends critically on the statistical validity of the model. Appropriate modelling of overdispersion can be critical for an accurate assessment of uncertainty for dynamic models [28,45–47].

In addition to the methodological points listed above, reproducibility and extendability are important practical considerations. Observational studies are not generally replicable in an experimental sense. However, the numerical conclusions should be readily reproducible from the observations. A substantial part of the value of a computational model is that it permits *in silico* experimentation of the modelled system. The authors should build and share a computational environment that not only reproduces published numbers but also facilitates future *in silico* experimentation. Subsequent research should readily be able to challenge the assumptions of the model in light of subsequent data. In practice, this requires that the scientists provide a free, open-source software environment within which the published analysis can readily be reproduced, modified and extended [5,60]. The development of a principled data analysis environment assists the researchers to explore their own models and data, and this environment should be shared as part of the publication process. In practice, this involves encapsulating data analysis within a software package that immerses the user in a documented environment where the models, methods and data used for the article can be readily experimented with. Trustworthy data analysis should be supported by unit testing and documentation, and the quality of this support should be one of the considerations in evaluating the data analysis. In other words, the article presenting the research should be part of a compendium [60]. The compendium for this article comprises the article source code, at https://github.com/jifanli/metapop_article, together with the software environment for the data analysis, provided by the R package at https://github.com/jifanli/metapoppkg.

In conclusion, the study of metapopulation dynamics will continue to benefit from advances in algorithms, software and data analysis methodologies. The models should undergo critical scrutiny to delineate their strengths and weaknesses, following evaluation procedures such as we have described in this paper. With due care, these models can unearth limitations in existing knowledge, investigate hypotheses that may extend our knowledge, and furnish us with valuable predictive tools.

## 4. Methods

### 4.1. Data

COVID-19 case reports, city population counts and the time-varying matrix of movement between cities were taken from [9]. Some erroneous numbers, revealed by our log-likelihood anomaly analysis, were subsequently modified as described in electronic supplementary material, section S1.

### 4.2. Model

All the mechanistic models under consideration have an SEAIR structure, as described in figure 1. Section S1, electronic supplementary material, provides a full mathematical representation of the SEAIR model. Briefly, the force of infection on the susceptible individuals for city u owing to symptomatic and asymptomatic individuals in city u is given by


(4.1) $$\mu_{S_{u}E_{u}}=\beta \left(\frac{I_{u}\left(t\right)+\mu A_{u}\left(t\right)}{P_{u}\left(t\right)}\right)d\Gamma_{u}/dt,$$

where β is the transmission rate, μ is the relative transmissibility of asymptomatic cases and $P_{u}$ is the city population. The gamma white noise process, $d\Gamma_{u}/dt$, allows for stochastic variation in the transmission rate [28]. The rate at which individuals move between each pair of cities is defined by a time-varying matrix based on high-resolution Tencent data from 2018, as described in electronic supplementary material, section S1.2. The basic reproductive number is given by


(4.2) $$R_{0}=\left(\alpha +\left(1-\alpha \right)\mu \right)D\beta ,$$

where D is the mean infectious period and α is the fraction of cases severe enough to be reported.

### 4.3. Likelihood evaluation and maximization

**Figure 4.**
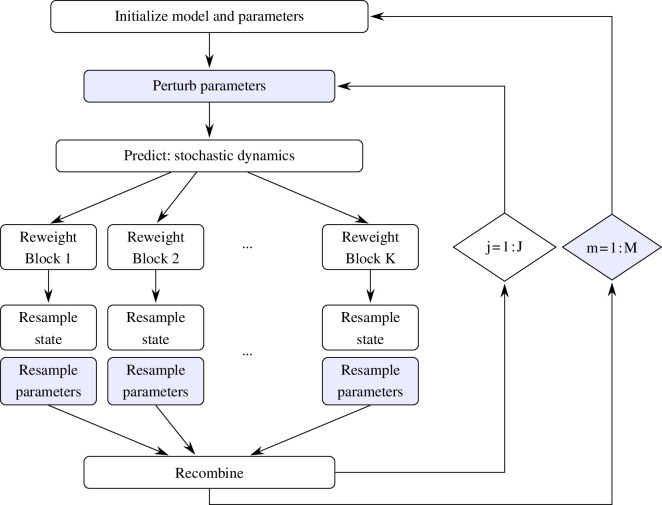
A flow diagram for an iterated block particle filter. The inner loop is a block particle filter, where the high-dimensional latent state is divided into K blocks. The J particles each contain a value for the entire latent state. The outer loop, together with the parameter perturbation and parameter resampling steps, comprises M filtering iterations for maximum likelihood parameter estimation.

The log-likelihood for the SpatPOMP models was calculated using the BPF. This log-likelihood was then maximized using an IBPF [50,51]. A diagram representing the IBPF algorithm is shown in figure 4. The inner loop, j=1,…,J, corresponds to the BPF applied to an extension of the model where parameters are perturbed by random noise, allowing the resampling step to provide Darwinian natural selection among the population of particles which favours parameter values consistent with the data. The outer loop, m=1,…,M, iterates this BPF procedure while decreasing the magnitude of the perturbations, which is theoretically guaranteed to guide the parameters towards a neighbourhood of the maximum likelihood estimate [22,51]. Further discussion of BPF and IBPF is in electronic supplementary material, section S4. Using this maximization procedure, we constructed confidence intervals by profile likelihood, employing Monte Carlo adjusted profiles [61,62] to correct for Monte Carlo variability. A single likelihood search took 8.5 h on a single computing core, using J=1000 particles and M=50 search iterations. Successful searches were continued for up to eight rounds of refinement until no further gain in likelihood was available. Multiple cores on a Linux cluster were used for Monte Carlo replication and parallel calculation of likelihood profiles.

### 4.4. Model criticism

A negative binomial autoregressive model was used to provide a non-mechanistic benchmark log-likelihood, as described in electronic supplementary material, section S2. This model was also used to construct benchmark conditional log-likelihoods for each separate observation. These, differenced from the corresponding SEAIR log-likelihoods, were used to define anomalies. The anomalies were explored to identify data points that were poorly explained by the model (electronic supplementary material, section S8). In the preliminary data analysis, these anomalies helped to identify some errors in the data, which were subsequently corrected (electronic supplementary material, section S1.3).

### 4.5. Software environment

The numerical work was carried out in R [63]. Models and data analysis methodology were developed in an R package, metapoppkg, which is additionally designed to assist the reproducibility and extendability of our results. Models in metapoppkg are implemented using spatPomp [64], which provides a general representation of SpatPOMP models extending the POMP model representation in pomp [23].

## Data Availability

The metapoppkg R package, containing the data and software used for this article, is available at [65] and archived at [66]. The manuscript source code is available at [67] and archived at [68]. Supplementary material is available online [69].
